# Study of the population genetic structure of *Opisthorchis*-like eggs in northern Thailand using mitochondrial genes

**DOI:** 10.1371/journal.pntd.0012445

**Published:** 2024-08-23

**Authors:** Picha Suwannahitatorn, Mathirut Mungthin, Ittisak Subrungruang, Lakhanawan Charoensuk, Nithikoon Aksorn, Saiwasan Buathong

**Affiliations:** 1 Department of Parasitology, Phramongkutklao College of Medicine, Ratchathewi, Bangkok, Thailand; 2 Department of Clinical Pathology, Faculty of Medicine Vajira Hospital, Navamindradhiraj University, Dusit, Bangkok, Thailand; University of Liverpool, UNITED KINGDOM OF GREAT BRITAIN AND NORTHERN IRELAND

## Abstract

**Background:**

*Opisthorchis-*like eggs are a public health problem in northern and northeastern Thailand. However, the genetic epidemiology and structure of these parasites in northern Thailand are unknown. Thus, this study investigated their population genetic structure using cytochrome c oxidase subunit 1 (*cox1*) and NADH dehydrogenase subunit 1 (*nad1*) nucleotide sequences.

**Methodology/Principal findings:**

A study was conducted in the hill tribe regions of Chiang Mai Province, northern Thailand. Internal transcribed spacer 2 polymerase chain reaction and restriction fragment length polymorphism were used to distinguish 205 positive feces samples for *Opisthorchis*-like eggs. The results showed that the prevalence of *O*. *viverrini* and *Haplorchis taichui* was 10.5% and 38.2%, respectively, and the co-infection rate was 37.2%. To determine the genetic structure of *O*. *viverrini* and *H*. *taichui* using *cox1* and *nad1* genes, genetic analysis was performed using 30 randomly chosen fecal samples for *Opisthorchis*-like eggs. Pairwise *F*_ST_ analysis indicated that *O*. *viverrini* and *H*. *taichui* displayed nonsignificant genetic differentiation within Chiang Mai Province and between interpopulations from different geographic areas. Moreover, within the intrapopulation in Chiang Mai Province, *cox1* presented higher gene flow than *nad1* in *O*. *viverrini*, while *nad1* demonstrated higher gene flow than *cox1* in *H*. *taichui*. The neutrality tests based on Fu’s *Fs* indicated population expansion and selective sweep from bottleneck or hitchhiking in *O*. *viverrini* and *H*. *taichui* populations, supported by haplotype network patterns. Phylogenetic tree analysis based on *cox1* and *nad1* revealed the monophyly of *O*. *viverrini* and *H*. *taichui* and genetic relationships with other isolates collected from Thailand, Lao People’s Democratic Republic (PDR), and Vietnam.

**Conclusions/Significance:**

This study investigated the molecular discrimination and genetic structure of *Opisthorchis*-like eggs in northern Thailand. The genetic information derived from this study could be associated with the background, molecular epidemiology, and disease severity of these parasites.

## Author summary

*Opisthorchis*-like egg infections are highly prevalent in northern and northeastern Thailand; however, their genetic epidemiology and structure in northern Thailand are unknown. Thus, this study examined the genetic structure of *Opisthorchis*-like eggs in the hill tribe population of Chiang Mai Province, northern Thailand, using mitochondrial genes [cytochrome c oxidase subunit 1 (*cox1*) and NADH dehydrogenase subunit 1 (*nad1*)]. A total of 205 positive fecal samples for *Opisthorchis*-like eggs were collected and examined using internal transcribed spacer 2 polymerase chain reaction and restriction fragment length polymorphism assays. Thirty fecal samples of *O*. *viverrini* and *Haplorchis taichui* were randomly selected for investigating population genetic structure using *cox1* and *nad1*. Pairwise *F*_ST_ analysis demonstrated nonsignificant genetic differentiation within the intrapopulation of Chiang Mai Province and interpopulations from different geographic areas. Gene flow estimation showed that *cox1* had higher gene flow than *nad1* in *O*. *viverrini*, whereas both genes showed low gene flow in *H*. *taichui*. Moreover, neutrality tests and haplotype networks revealed a population expansion from the bottleneck or selective sweep. Additionally, the phylogenetic trees of *O*. *viverrini* and *H*. *taichui* revealed monophyletic groups and were genetically similar to isolates in other regions in Thailand, Lao PDR, Cambodia and Vietnam.

## Introduction

In Thailand, food-borne trematode infections (especially human liver fluke [*Opisthorchis viverrini*] and minute intestinal flukes [MIFs] in the northern and northeastern regions) remain a public health problem [1,2]. *O*. *viverrini* infections cause opisthorchiasis. Most clinical manifestations of opisthorchiasis are asymptomatic; however, chronic infection can lead to cholangiocarcinoma (CCA), which is diagnosed at a late stage, resulting in poor prognosis and difficult treatment [3–8]. Thailand has the highest global prevalence of CCA, especially in the northeastern region (89 per 100,000 for females and 129 per 100,000 for males) [9]. MIF infections are often asymptomatic, but heavy infections can cause intestinal irritation and inflammation, reducing nutrient and fluid absorption. Severe symptoms occur when parasite eggs pass through the intestinal wall into the bloodstream, damaging the internal organs [10]. Currently, *O*. *viverrini* and MIF infections are diagnosed by detecting parasite eggs in feces under a light microscope using the Kato–Katz technique or formalin-ethyl acetate concentration technique (FECT) [11,12]. However, *O*. *viverrini* eggs are difficult to distinguish from MIF eggs under a light microscope because they have similar size and shape; therefore, they are identified as *Opisthorchis*-like eggs [1,13–15]. The Chinese liver fluke (*Clonorchis sinensis*) was reported to have a prevalence of 23% among residents in central Thailand [16], and chronic infection with this parasite can lead to CCA. However, there has only been one report on the prevalence of *C*. *sinensis* in Thailand. Despite evidence showing the distribution of the freshwater snail *Melanoides tuberculata*, which may serve as the first intermediate host of *C*. *sinensis* in Thailand that could support its life cycle, comprehensive studies on the prevalence of this parasite in Thailand are lacking [17]. Additionally, *M*. *tuberculata* is distributed in the northern, eastern, and central regions of Thailand [17], suggesting that the prevalence of this parasite should also be monitored in northern Thailand. Currently, molecular-based polymerase chain reaction (PCR) approaches are commonly used to differentiate *Opisthorchis*-like eggs. In these methods, internal transcribed spacers 1 and 2 (ITS2) [16,18,19] and mitochondrial genes (e.g., cytochrome c oxidase subunit 1 [*cox1*] [20,21], NADH dehydrogenase subunit 1 [*nad1*] [21,22], and NADH dehydrogenase subunit 2 [*nad2*] [23,24]) are used as molecular targets for discrimination. Moreover, multilocus enzyme electrophoresis [25,26], random amplified polymorphic DNA [27,28], microsatellite markers [29,30], and mitochondrial sequencing [15,22,31] have been used to examine the genetic structure and diversity of *O*. *viverrini*. Mitochondrial DNA sequences are widely used to analyze the genetic structures of parasites such as *Fasciola hepatica* [32,33], *Paragonimus westermani* [34,35], and *Schistosoma* spp. [36–38] because of their high mutation rates and maternal inheritance characteristics, allowing for a comprehensive understanding of their genetic variation and population structure. Thus, *cox1* and *nad1* could be used for investigating the genetic structure and diversity among *O*. *viverrini* populations in northern Thailand, where mitochondrial DNA sequence data are lacking.

In northern Thailand, the prevalence of MIF infections, especially *Haplorchis taichui*, is higher than that of *O*. *viverrini* infections. According to a previous study, the prevalence of *H*. *taichui* in Nan and Lampang Provinces was 70.8% and 69.0%, respectively, whereas *O*. *viverrini* was not found [39]. In another study that performed stool examinations of residents in Chiang Mai Province, northern Thailand, the prevalence rates of *O*. *viverrini* and *H*. *taichui* were 10.5% and 38.2%, respectively, and the co-infection rate was 37.2% [2]. Conversely, high prevalence of *O*. *viverrini* was not observed in several endemic locations of northern Thailand, which uncorrelated to CCA incidence, and MIFs infections do not produce CCA in this region [39]. Since a high prevalence of *Opisthorchis*-like egg infections have been reported among hill tribe residents of Chiang Mai, northern Thailand, the Ministry of Public Health of Thailand and collaborating health agencies launched a national strategic plan to control liver fluke infections and CCA, with the goal of reducing the prevalence of these parasites and CCA in northern Thailand [2]. However, the genetic structure of *Opisthorchis*-like eggs in these endemic areas is unknown. Therefore, the present study aimed to investigate the presence and genetic structure of *Opisthorchis*-like eggs collected from fecal samples of hill tribe residents in Chiang Mai Province, northern Thailand, using mitochondrial genes (*cox1* and *nad1*). Using fecal samples containing *Opisthorchis*-like eggs can provide a representative sample of the parasite population within infected individuals and will reflect the overall genetic diversity present in the parasite population within the host population. Moreover, pooled parasite eggs from fecal samples of infected individuals enables more efficient resource utilization by reducing the number of individual samples collected and invasive processes required for each sample. The findings of this study are crucial for the development of disease prevention and long-term control strategies, surveillance efforts, and understanding the diversity and evolution of these parasites in northern Thailand.

## Materials and methods

### Ethics statement

The research protocol for this study was reviewed and approved by the Institutional Review Board of the Faculty of Medicine Vajira Hospital. The study was granted an exemption in accordance with international guidelines for the protection of human research participants, including the Declaration of Helsinki, The Belmont Report, CIOMS Guidelines, the International Conference on Harmonization in Good Clinical Practice (ICH-GCP), and 45 CFR 46.101(b), under approval number COE: 19/2021X. As the fecal samples used in this study were collected as part of routine public health surveillance activities under the national strategic plan to control liver fluke infections and cholangiocarcinoma (CCA) in northern Thailand, implemented by the Ministry of Public Health in collaboration with other health agencies, these samples were anonymized and used exclusively for public health research purposes. Therefore, individual consent forms were not required.

### Stool collection

A cross-sectional study was conducted on hill tribe communities located in Chaing Mai Province, northern Thailand. A total of 205 positive samples for *Opisthorchis*-like eggs were obtained from the stool bank of the Ministry of Public Health and collaborating health agencies. These fecal samples were collected from 94 infected individuals in Doi Tao District, 67 in Mae Tang District, and 47 in Chiang Dao District, as part of the national strategic plan to control liver fluke infections and CCA in 2020. However, these samples do not contain demographic data. The three districts are primarily rural in nature and surrounded by mountains and forests. Furthermore, the residents share a deep-rooted connection to the land and nature, with agriculture (especially rice cultivation) serving as a major economic activity. Furthermore, the residents include ethnic Thai individuals and various hill tribe communities such as Karen, Hmong, and Lahu. These people have a diverse culture; they speak their own languages and have preserved their ancient customs [40].

### DNA extraction of *Opisthorchis*-like eggs from fecal samples

Each *Opisthorchis*-like egg-positive sample was sedimented using phosphate buffered saline (PBS) ethyl acetate concentration technique in accordance with Buathong’s protocols [15]. Briefly, 200 μL of each sample was thoroughly homogenized by mixing with 1.4 mL of ATL tissue lysis solution and breaking up the eggs through PBS incubation method and five freeze–thaw cycles using liquid nitrogen at 100°C. Then, DNA extraction was performed using the QIAamp DNA Stool Mini Kit (Qiagen) in accordance with the manufacturer’s instructions. Finally, the DNA was eluted to a volume of 50 μL with elution buffer and used as a template for PCR assays.

### Discrimination of *Opisthorchis*-like eggs using PCR and restriction fragment length polymorphism (RFLP) assays

A total of 205 *Opisthorchis*-like egg-positive samples were subjected to PCR assay using RTFluke primers, which were designed by Traub et al. [16] and could amplify the ITS2 regions of human liver flukes and MIFs in the Heterophyidae family. PCR amplifications were performed in a total volume of 25 μL using a DNA template, 10 pmol of each RTFluke primer, and 1X KAPA2G Fast HotStart ReadyMix with dye (Roche). The PCR products were amplified using the Mastercycler personal (Bio-Rad). ITS2-PCR assay was performed in accordance with Traub et al.’s protocols [16]. The amplicons of *O*. *viverrini*, *C*. *sinensis*, and *H*. *taichui* were 375, 381, and 526-bp, respectively. To differentiate between *O*. *viverrini* and *C*. *sinensis*, the 375 and 381-bp PCR products were subjected to discrimination analysis using the *Fau*I restriction enzyme (New England Biolabs) based on RFLP. After digestion at 55°C for 6 h, the PCR products for *O*. *viverrini* comprised 129 and 247-bp fragments, whereas *C*. *sinensis* yielded an undigested amplicon [15]. The results of ITS2-PCR and RFLP assays for discriminating *Opisthorchis*-like eggs revealed only the presence of *O*. *viverrini* and *H*. *taichui*. Therefore, genetic analysis of *cox1* and *nad1* was performed for *O*. *viverrini* and *H*. *taichui*.

### Evaluation of PCR assays to detect *O*. *viverrini* and *H*. *taichui* in fecal samples based on *cox1* and *nad1*

A subset of 30 positive samples, confirmed through ITS2-PCR for *O*. *viverrini* and *H*. *taichui*, was randomly selected for further analysis using COX1- and NAD1-PCR assays and genetic analysis. The PCR-based assay targeting *cox1* of *O*. *viverrini* was evaluated using primers designed by Bauthong et al. [21]. To evaluate of *nad1* of *O*. *viverrini*, the COX1-PCR assay of *O*. *viverrini* was performed in a total volume of 25 μL, comprising a DNA template, 10 pmol of each primer, and 1X KAPA2G Fast HotStart ReadyMix with dye (Roche). New primers with greater sensitivity were designed using Primer3web version 4.1.0 (https://primer3.ut.ee/) and used in this study. The NAD1*-*PCR assay of *O*. *viverrini* was performed in a total volume of 25 μL, comprising a DNA template, 10 pmol of each primer (OVNADI-F, 5′-TCAG GTACGCAGGTGGTT TG-3′; OVNADI-R, 5′-CCTTCGCAAG GT TAACAGCC-3′), and 1X KAPA2G Fast HotStart ReadyMix with dye (Roche). The PCR products of *cox1* of *O*. *viverrini* were amplified in the Mastercycle personal (Bio-Rad). The PCR assay of OVNADI primers was conducted as follows: predenaturation at 95°C for 5 min, followed by 30 cycles of denaturation at 95°C for 45 s, annealing at 60°C for 45 s, extension at 72°C for 1 min, final extension at 72°C for 10 min, and holding at 12°C.

To investigate mitochondrial genes in *H*. *taichui*, the primers and optimal PCR assays of *cox1* conducted by Thaenkham et al. were used [22]. The COX1-PCR assay for *H*. *taichui* was conducted in a total volume of 25 μL, comprising a DNA template, 10 pmol of each primer, and 1X KAPA2G Fast HotStart ReadyMix with dye (Roche). The primers used for detecting *nad1* of *H*. *taichui* were designed using Primer3web version 4.1.0 (https://primer3.ut.ee/). The primer sequences for the NAD1-PCR assay of *H*. *taichui* were as follows: HTNAD1-F, 5′GGTGGCTAGACACT CAGAGC-3′; HTNAD1-R, 5′-AGCCCCCAAAGCTAACATCC-3′. PCR assay was performed in a total volume of 25 μL, comprising a DNA template, 10 pmol of each primer, and 1X KAPA2G Fast HotStart ReadyMix with dye (Roche). The optimal reaction conditions for the NAD1-PCR assay of *H*. *taichui* were as follows: predenaturation at 95°C for 5 min, followed by 30 cycles of denaturation at 95°C for 45 s, annealing at 60°C for 45 s, extension at 72°C for 1 min, final extension at 72°C for 10 min, and holding at 12°C.

The PCR products were run on a 2% agarose gel and visualized using the Molecular Imager Gel Doc XR+ Imaging System (Bio-Rad). Thereafter, the PCR products of *cox1* and *nad1* from *O*. *viverrini* and *H*. *taichui* were individually excised from agarose gel and placed into 2 mL tubes for gel extraction and DNA purification using the QIAquick Gel Extraction Kit (Qiagen) in accordance with the manufacturer’s protocols. Subsequently, the samples were sent to U2Bio (Thailand) for DNA sequencing. The DNA sequences obtained in this study were aligned using the ClustalW program incorporated within the MEGA-X software [41], which allows for precise alignment and comparison. After alignment, the sequences were compared with a comprehensive database of known nucleotide sequences using the Basic Local Alignment Search Tool to validate species identification and determine species origin. To obtain accession numbers of each haplotype, the nucleotide sequences of *cox1* and *nad1* from *Opisthorchis-*like eggs obtained in this study were submitted to the GenBank database. Subsequently, the accession numbers of *cox1* and *nad1* from *Opisthorchis*-like eggs were utilized for phylogenetic tree and haplotype network analysis.

### Sequence analysis of *cox1* and *nad1* from *O*. *viverrini* and *H*. *taichui* to determine population genetic structure

The population genetic structure of *O*. *viverrini* and *H*. *taichui* was determined using a total positive sample from COX1- and NAD1-PCR assays. The *cox1* and *nad1* nucleotide sequences of *O*. *viverrini* and *H*. *taichui* were genetically analyzed for haplotype diversity (Hd) values, segregation sites across populations (S), and nucleotide diversity (Pi) using DnaSP version 5.10.1 [42]. The genetic differentiation in *O*. *viverrini* and *H*. *taichui* populations was evaluated using pairwise *F*_ST_ values estimated from DnaSP version 5.10.1 [42]. *F*_ST_ values between 0.0 and 0.05 imply small genetic differences between populations (or some gene flow), values between 0.05 and 0.15 indicate moderate genetic differences, and values greater than 0.15 suggest high genetic differentiation and low (or no) gene flow. The gene flow between *O*. *viverrini* and *H*. *taichui* populations was calculated using *N*_*e*_*m* = (1/*F*_ST_− 1)/4 [43]. The genetic differentiation and gene flow of *cox1* within the *O*. *viverrini* intrapopulation from Chiang Mai Province were analyzed and compared with the interpopulations from northeastern Thailand (MDM-5, BR-2, MD-5, SKPK18M-3, SKNH-5, H5, HH22-1, VTNK), eastern Thailand (SK), Lao PDR (VT, KM-1, SV-4, accession number JF739555), and Vietnam (BD1). Additionally, the genetic differentiation and gene flow of *cox1* in *O*. *viverrini* from 36 isolates, comprising 15 recently identified haplotypes from cats isolated in northeastern Thailand (OM424139–OM424174), [44] were analyzed and compared with haplotypes obtained from Chiang Mai Province to determine genetic relationship and transmission dynamics. Furthermore, the genetic differentiation and gene flow of *nad1* within the *O*. *viverrini* intrapopulation in Chiang Mai Province were evaluated and compared with the interpopulations from different geographic areas, including northeastern Thailand (KLp, KS, KBp, KPv, KK, DQ119551), eastern Thailand (SK/3), Vietnam (BD, OvL, DL3, QN, PY3, OvPY3), Lao PDR (VT/1, SV/2, KM/1, JF739555, CP/14), and Cambodia (KD/4). For *H*. *taichui*, the genetic differentiation and gene flow of *cox1* were investigated within the intrapopulation from Chiang Mai Province and compared with the interpopulations across geographically distinct endemic regions, including northern Thailand (CR1, CR3, CR4, PY7), central Thailand (CH1), Vietnam (HG4, QT3, QT6, QT15, TH3, TH5, TH11, YB, KC404636), Lao PDR (BK1, BK3, SV1, CP1), and the Philippines (PHL1). Furthermore, the genetic differentiation and gene flow of *nad1* in *H*. *taichui* were investigated and compared with the *H*. *taichui* interpopulations from other endemic locations, including Vietnam (HTAQT3, QT3) and Korea (NC_022433). Moreover, the neutrality tests of *cox1* and *nad1* of *O*. *viverrini* and *H*. *taichui* obtained in this study were analyzed for the historical dynamics of the population using Fu’s *Fs* and Tajima’s *D* tests from Arlequin version 3.1 [45]. Fu’s *Fs* test is a statistical method that examines the allele frequency distribution in a population, whereas Tajima’s *D* test evaluates the difference between the number of segregating sites and the average number of pairwise differences in a DNA sequence alignment. A significantly negative value of Fu’s *Fs* or Tajima’s *D* test suggests an excess of rare alleles, which may indicate recent population expansion, selective sweeps, or background selection. Conversely, a significantly positive value may indicate population reduction, balanced selection, or population subdivision. Furthermore, extensions of Fu’s *Fs* and Tajima’s *D* tests, known as Fu and Li’s *F* and Fu and Li’s *D* tests, were performed using DnaSP version 5.10.1 [42]. These tests are useful for detecting population expansions or bottlenecks. A significantly negative value of Fu and Li’s *F* or Fu and Li’s *D* test suggests an excess of singleton mutations, indicating population expansion or positive selection. By contrast, a significantly positive value suggests a deficit of singleton mutations, which may indicate population reduction or purifying selection.

### Phylogenetic tree and haplotype network analysis of *O*. *viverrini* and *H*. *taichui* using *cox1* and *nad1*

The nucleotide sequences of *cox1* and *nad1* in *O*. *viverrini* and *H*. *taichui* were analyzed using the ClustalW program in the MEGA-X software. Maximum likelihood phylogenetic trees were constructed using the Tamura 3-parameter in the MEGA-X software with 1,000 replications of bootstrapping values based on *cox1* and *nad1* of *O*. *viverrini* and *H*. *taichui*. [46]. The reference sequences used in the *O*. *viverrini* and *H*. *taichui* phylogenetic trees were obtained from the GenBank database. Haplotype networks were generated using the *cox1* and *nad1* nucleotide sequences of *O*. *viverrini* and *H*. *taichui*. The haplotype networks were computed using a median-joining network algorithm from Network 10, which could be accessed at the following URL: http://www.fluxus-engineering.com. Moreover, the reference isolates of *O*. *viverrini* and *H*. *taichui* were retrieved from the GenBank database.

The phylogenetic trees and haplotype networks of *cox1* in *O*. *viverrini* were constructed using haplotypes from Chiang Mai Province (CM1–CM9), northeastern Thailand (MDM-5, BR-2, MD-5, SKPK18M-3, SKNH-5, H5, HH22-1, VTNK, NP, SSK), eastern Thailand (SK), Lao PDR (VT, KM-1, SV-4, accession number JF739555), and Vietnam (BD1). Additionally, the *cox1* sequences of 15 *O*. *viverrini* haplotypes obtained from cats (OM424139–OM424174) [44] were included in the phylogenetic analysis and haplotype network. Furthermore, the phylogenetic trees and haplotype networks of *nad1* in *O*. *viverrini* comprised haplotypes from Chiang Mai Province, northeastern Thailand (KLp, KS, KBp, KPv, KK, DQ11955), eastern Thailand (SK/3), Vietnam (BD, OvL, DL3, QN, PY3, OvPY3), Lao PDR (VT/1, SV/2, KM/1, JF739555, CP/14), and Cambodia (KD/4). For the phylogenetic trees and haplotype networks of *cox1* in *H*. *taichui*, haplotypes isolated from Chiang Mai Province, northern Thailand (CR1, CR3, CR4, PY7), central Thailand (CH1), Vietnam (HG4, QT3, QT6, QT15, TH3, TH5, TH11, YB, KC404636), Lao PDR (BK1, BK3, SV1, CP1), and the Philippines (PHL1) were analyzed. Moreover, the phylogenetic trees and haplotype networks of *nad1* in *H*. *taichui* were generated using haplotypes from Chiang Mai Province, Vietnam (HTAQT3 and QT3), and Korea (NC_022433).

## Results

### Prevalence of *Opisthorchis-like* eggs using ITS2-PCR and RFLP assay in fecal samples

The ITS2-PCR-assay of 205 fecal samples showed a sensitivity of 82.9% (168/205) for detecting *Opisthorchis*-like eggs in fecal samples. After amplifying with RTFluke primers, the ITS2-PCR products of O. *viverrini*, *C*. *sinensis*, and *H*. *taichu*i were 375, 381, and 526-bp, respectively. The RFLP profiles of *Opisthorchis*-like eggs revealed that the prevalence of a single infection of *O*. *viverrini* and *H*. *taichui* was 2.4% (5/168) and 50.7% (104/168), respectively. Additionally, the prevalence of mixed infections with *O*. *viverrini* and *H*. *taichui* was 28.8% (59/168). Thus, the overall prevalence of *O*. *viverrini* and *H*. *taichui* was 31.2% (64/168) and 79.5% (163/168), respectively (Table 1).

**Table 1:** Proportion of *Opisthorchis-*like egg infections in fecal samples as determined by ITS2-PCR assay (n = 205).

Parasites	Number of positive fecal samples	% of positive fecal samples
*O*. *viverrini* only	5	2.4
*H*. *taichui* only	104	50.7
Mixed infection of *O*. *viverrini* and *H*. *taichui*	59	28.8
Overall presence of *O*. *viverrini*	64	31.2
Overall presence of *H*. *taichui*	163	79.5

The analysis of *Opisthorchis*-like egg infections by district revealed distinct prevalence rates. In Doi Tao District, the rate of single infection with *O*. *viverrini* was 5.3% (5/94), while no cases were found in Mae Tang and Chiang Dao Districts. Notably, single infections with *H*. *taichui* were higher in Doi Tao District, occurring in 50% (47/94) of the samples. Additionally, mixed infections involving both *O*. *viverrini* and *H*. *taichui* were observed in 36.2% (34/94) of the samples from this district. In Mae Tang District, single infections with *H*. *taichui* were prevalent at 56.7% (38/67), while mixed infections of *H*. *taichui* and *O*. *viverrini* occurred at 25.4% (17/67). In Chiang Dao District, the prevalence of single infections with *H*. *taichui* was 46.8% (22/47), whereas mixed infections with both *O*. *viverrini* and *H*. *taichui* were observed in 27.7% (13/47). Overall, the prevalence analysis by district revealed that single infections with *H*. *taichui* and mixed infections with both *O*. *viverrini* and *H*. *taichui* were common across all three districts. Furthermore, *C*. *sinensis* was not detected in this study.

### Evaluation of COX1- and NAD1-PCR assays to detect *O*. *viverrini* and *H*. *taichui* using fecal samples

**Fig 1:**
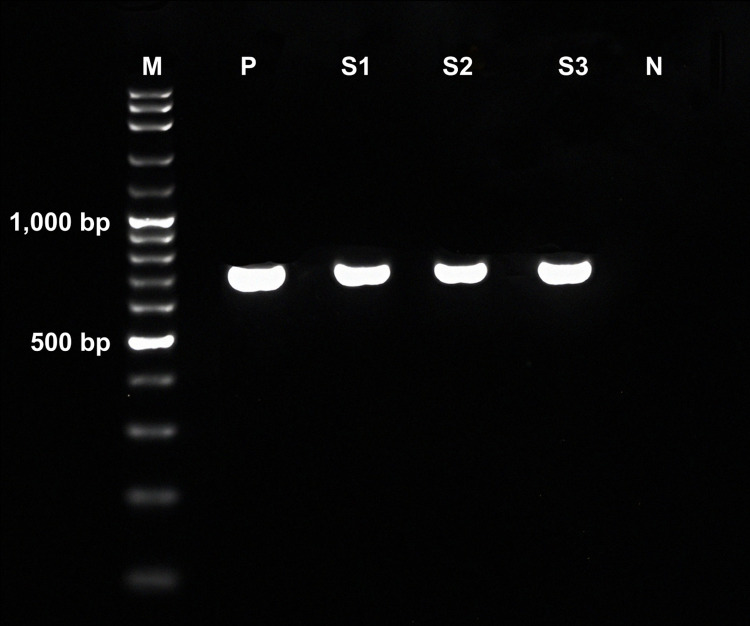
PCR products of *nad1* in *Opisthorchis viverrini* after amplification with OVNADI primers. The PCR products of NAD1-PCR assay of *O*. *viverrini* were 744-bp. M is the 100-bp DNA ladder, P is the positive control, S1–S3 show the NAD1-PCR products of *O*. *viverrini* from fecal samples, and N is the negative control.

Thirty positive samples for *O*. *viverrini* were randomly selected from a total of 64 samples identified across three districts. These selected 30 samples for *O*. *viverrini* were then used to evaluate the COX1- and NAD1-PCR assays and for subsequent genetic analysis. The sensitivities of COX1- and NAD1-PCR assays of *O*. *viverrini* in 30 positive fecal samples were 86.7% (26/30) and 66.7% (20/30), respectively. After amplification, the PCR products of *cox1* and *nad1* in *O*. *viverrini* were 504 and 744-bp (Fig 1), respectively. Moreover, COX1-PCR assay showed higher sensitivity than NAD1-PCR assay for detecting *O*. *viverrini* in fecal samples. In addition, the OVNADI primers used in the NAD1-PCR assay for amplifying the *nad1* gene in *O*. *viverrini* did not cross-react with other intestinal parasites in fecal samples, including *Ascaris lumbricoides*, hookworm, *Taenia* spp., *Trichuris trichiura*, *H*. *taichui*, *Giardia intestinalis*, *Blastocystis* sp., and *Entamoeba coli*.

**Fig 2:**
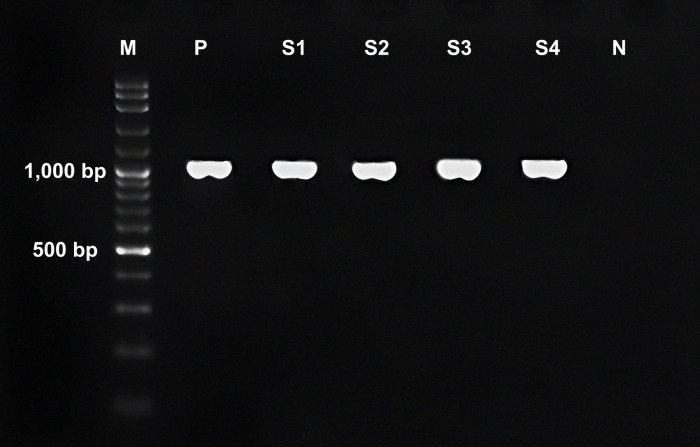
PCR products of *nad1* in *Haplorchis taichui* after amplification with HTNAD1 primers. The PCR products of NAD1-PCR assay of *H*. *taichui* were 1,040-bp. M is the 100-bp DNA ladder, P is the positive control, S1–S4 show the NAD1-PCR products of *H*. *taichui* from fecal samples, and N is the negative control.

Similarly, 30 positive samples of *H*. *taichui* were randomly selected from a total of 164 positive samples collected across three districts. These selected *H*. *taichui* positive samples were subsequently subjected to COX1- and NAD1-PCR assays for genetic analysis. The sensitivity of COX1- and NAD1-PCR assays for detecting *H*. *taichui* in fecal samples was 96.7% (29/30) and 83.3% (26/30), respectively. The PCR products of *cox1* and *nad1* in *H*. *taichui* were 375 and 1,040-bp (Fig 2), respectively. The HTNAD primers used in NAD1-PCR assay were first developed for detecting *nad1* in *H*. *taichui* in fecal samples, and no cross-reactions with other intestinal parasites were noted, including *O*. *viverrini*. Furthermore, ITS2-PCR assay exhibited greater sensitivity than COX1- and NAD1-PCR assays for detecting *O*. *viverrini* and *H*. *taichui* in fecal samples. However, the difference was not statistically significant (*p* > 0.05; 95% confidence interval).

### Population genetic structure analysis of *cox1* and *nad1* in *O*. *viverrini* and *H*. *taichui*

Genetic analysis was conducted on fecal samples of *O*. *viverrini* collected from Chiang Mai Province. The investigation of 26 *cox1* and 20 *nad1* nucleotide sequences revealed 9 *cox1* haplotypes (CM1–CM9; accession numbers: OR514397–OR514405) and 10 *nad1* haplotypes (CMNMU1–CMNMU10; accession numbers: OR528872–OR528881). Moreover, *cox1* and *nad1* in *O*. *viverrini* had 7 and 11 polymorphism sites (S) and presented haplotype diversity (Hd) values of 0.738 and 0.837, respectively. In addition, the nucleotide diversity (Pi) values of *cox1* and *nad1* in *O*. *viverrini* were 0.00244 and 0.00482, respectively (Table 2). From pairwise *F*_ST_ analysis, *O*. *viverrini* within the intrapopulation in Chiang Mai Province revealed low genetic variation in *cox1* (*F*_ST_ = 0.03704) and high genetic variation in *nad1* (*F*_ST_ = 0.51020). However, the population of *O*. *viverrini* in this region showed no significant genetic difference (all *p* > 0.05). Gene flow estimation of *O*. *viverrini* showed that *cox1* exhibited high gene flow (*N*_*e*_*m* = 5.75), whereas *nad1* exhibited low gene flow (*N*_*e*_*m* = 0.396) (Table 3).

**Table 2:** *cox1* and *nad1* nucleotide analyses of *O*. *viverrini* and *H*. *taichui* collected from Chiang Mai Province, northern Thailand.

Parasite’s name	Gene	Number of samples	Number of haplotypes	S	Hd	Pi
*O*. *viverrini*	*cox1*	26	9	7	0.738	0.00244
	*nad1*	20	10	11	0.837	0.00482
*H*. *taichui*	*cox1*	29	9	10	0.640	0.00310
	*nad1*	25	15	28	0.937	0.00384

**Table 3:** Genetic differentiation estimation and neutrality tests of *cox1* and *nad1* of *O*. *viverrini* and *H*. *taichui* collected from Chiang Mai Province, northern Thailand.

Parasite’s name	Gene	*F*_ST_ value		*N*_*e*_ *m*		Neutrality tests			
		Intrapopulation	Interpopulations	Intrapopulation	Interpopulations	Fu’s *Fs*	Tajima’s *D*	Fu and Li’s *D*	Fu and Li’s *F*
*O*. *viverrini*	*cox1*	0.03704	0.33040	5.75	0.51	-8.09309*	-1.35941	-1.27936	-1.45002
	*nad1*	0.51020	0.47480	0.19	0.28	-3.80761*	0.40504	0.54040	0.59026
*H*. *taichui*	*cox1*	0.28000	0.36621	0.64	0.43	-7.08967*	-1.00013	-1.01869	-1.03378
	*nad1*	0.20879	0.24133	0.95	0.79	-11.7077*	-1.86167*	-1.9796	-2.23173*

* a statistically significant test result (p ≤ 0.05)

The *cox1* between *O*. *viverrini* interpopulations in Chiang Mai Province (CM1–CM9), northern Thailand (LP-1 and LP-5), northeastern Thailand (MDM-5, BR-2, MD-5, SKPK18M-3, SKNH-5, H5, HH22-1, VTNK, NP, SSK), eastern Thailand (SK), Lao PDR (VT, KM-1, SV-4, accession number JF739555), Vietnam (BD1), and 15 cat haplotypes (CAT1, CAT2, CAT3, C4, C24, C13-6, C16, C40-1, C49, C10-6, C14-2, C51, C10-4, C19, C10-1) exhibited high genetic variation (*F*_ST_ = 0.33040) and low gene flow (*N*_*e*_*m* = 0.51), indicating limited genetic exchange between them. Meanwhile, *nad1* in *O*. *viverrini* collected from Chiang Mai Province (CMNMU1–CMNMU10), northeastern Thailand (KLp, KS, KBp, KK, KPv, DQ119551), eastern Thailand (SK/3), Vietnam (BD, OvL, DL3, QN, PY3, OvPY3), Cambodia (KD/4), and Lao PDR (VT/1, SV/2, KM/1, JF739555, CP/14) displayed high genetic variation (*F*_ST_ value = 0.47480) and low gene flow (*N*_*e*_*m* = 0.28). However, the genetic differentiation of *cox1* and *nad1* between *O*. *viverrini* interpopulations from various geographic locations, including Chiang Mai Province, northern Thailand, northeastern Thailand (including of cat haplotypes), eastern Thailand, Vietnam, Cambodia, and Lao PDR, was not significant (all *p* > 0.05). Among the interpopulations of *O*. *viverrini* from various geographic endemic regions, *cox1* showed a higher gene flow (*N*_*e*_*m* = 0.51) compared to *nad1* (*N*_*e*_*m* = 0.28). The neutrality tests of *cox1* and *nad1* in *O*. *viverrini* in Chiang Mai Province based on Fu’s *Fs* exhibited statistically significant results (−8.09309 and −3.80761, respectively; all *p* < 0.05), indicating that the intrapopulation is growing and hitchhiking as a consequence of population expansion and selective sweep [47]. However, Tajima’s *D*, Fu and Li’s *D*, and Fu and Li’s *F* tests did not yield statistically significant results (all *p >* 0.01) (Table 3).

Twenty-nine *cox1* and 26 *nad1* nucleotide sequences of *H*. *taichui* were analyzed and provided 9 haplotypes (CMNMU1–CMNMU9; accession numbers: OR514388–OR514396) and 15 haplotypes (CMHT1–CMHT15; accession numbers: OR528882–OR528896), respectively. Haplotype analysis of *H*. *taichui* revealed 10 and 28 polymorphic sites from *cox1* and *nad1*, respectively. The haplotype diversity values of *cox1* and *nad1* in *H*. *taichui* were 0.640 and 0.937, respectively. Additionally, the nucleotide diversity values of *cox1* and *nad1* were 0.00314 and 0.00384, respectively, indicating low nucleotide diversity (Table 2). Pairwise *F*_ST_ analysis of *cox1* and *nad1* in *H*. *taichui* within the intrapopulation obtained from Chiang Mai Province exhibited high genetic differentiation with *F*_ST_ values of 0.28000 and 0.20879, respectively, but the difference was not significant (all *p* > 0.05). In addition, the study indicated that *cox1* and *nad1* within the intrapopulation of *H*. *taichui* exhibited low gene flow, with *N*_*e*_*m* values of 0.64 and 0.95, respectively (Table 3). The investigation of genetic differentiation in *H*. *taichui* using *cox1* across geographically distinct endemic regions, including Chiang Mai (CMNMU1–CMNMU9), northern Thailand (CR1, CR3, CR4, PY7), central Thailand (CH1), Vietnam (HG4, QT3, QT6, QT15, TH3, TH5, TH11, YB, KC404636), Lao PDR (BK1, BK3, SV1, CP1), and the Philippines (PHL1), revealed high genetic variation (*F*_ST_ = 0.36621, *p* > 0.05). High genetic variation was also observed in *nad1* (*F*_ST_ = 0.24133, *p* > 0.05). of *H*. *taichui* between interpopulations from Chiang Mai, Vietnam (HTAQT3, QT3), and Korea (NC_022433). However, the genetic differentiation of *cox1* and *nad1* in *H*. *taichui* did not show statistically significant results. Moreover, the gene flow of *cox1* (*N*_*e*_*m* = 0.43) and *nad1* (*N*_*e*_*m* = 0.79) in *H*. *taichui* between interpopulations in these areas was negligible (Table 3). Fu’s *Fs* test demonstrated negative statistical significance for *cox1* and *nad1* in *H*. *taichui* (−7.08967 and −11.7077, respectively; all *p* < 0.05), indicating population growth from a bottleneck or selective sweep (Table 3). Moreover, the statistically significant results of Fu and Li’s *F* test revealed evidence of background selection of *nad1* in *H*. *taichui* within intrapopulation of Chiang Mai Province.

### Phylogenetic tree analysis of *O*. *viverrini* and *H*. *taichui* based on *cox1* and *nad1*

**Fig 3:**
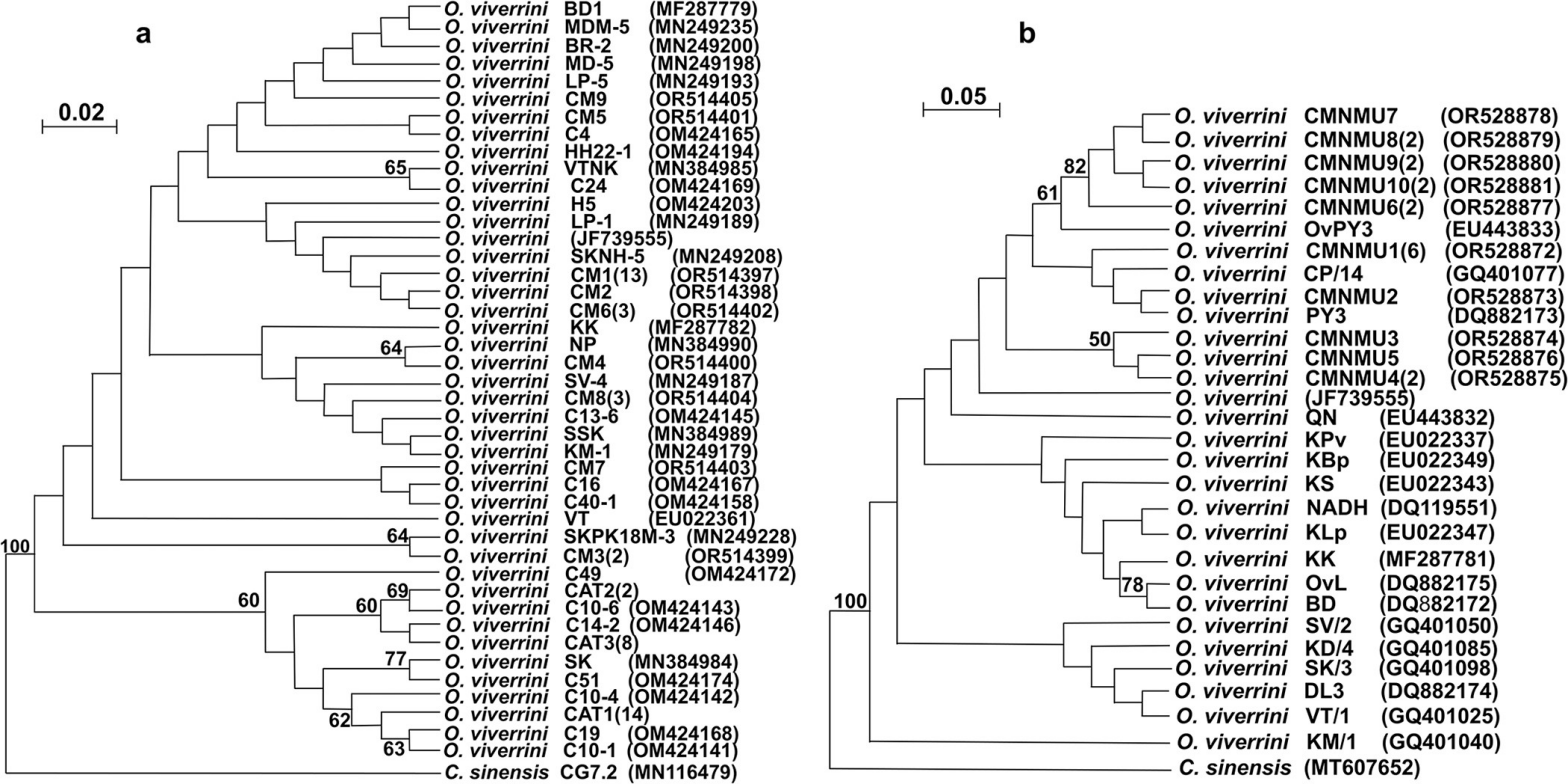
**Maximum likelihood phylogenetic trees based on *cox1* (a) and *nad1* (b) of *Opisthorchis viverrini*.** (a) The *cox1* phylogenetic tree, 405-bp nucleotide sequences without gaps, and nine haplotypes (CM1–CM9) obtained from Chiang Mai Province were aligned. The reference *cox1* isolates were from northern Thailand (LP-1, LP-5), northeastern Thailand (MDM-5, BR-2, MD-5, SKPK18M-3, SKNH-5, H5, HH22-1, VTNK, NP, SSK), Lao PDR (VT, KM-1, SV-4, JF739555), Vietnam (BD1), and 15 cat haplotypes. Haplotype CAT1 comprised C3-5, C8-1, C13-2, C14-5, C21-2, C29-4, C38-2, C39-2, C40B, C43-1, C7, C25, C46, and C50. Haplotype CAT2 comprised C15B-3 and C43-2. Haplotype CAT3 comprised C14-4, C27-6, C35A-1, C38-1, C39-3, C40-3, C43-5, and C1. Other cat haplotypes included C4, C24, C13-6, C16, C40-1, C49, C10-6, C14-2, C51, C10-4, C19, and C10-1. (b) The phylogenetic tree of *nad1* was constructed from 668-bp nucleotide sequences without gaps and 10 haplotypes (CMNMU1–CMNMU10) isolated from Chiang Mai Province. The reference isolates from Thailand (KLp, KS, KBp, KPv, KK, DQ119551), Lao PDR (VT/1, SV/2, KM/1, JF739555, CP/14), Vietnam (BD, OvL, DL3, QN, PY3, OvPY3), and Cambodia (KD/4) were used. The nodes showed 1,000 replication (bootstrap) percentages. More than 50% were displayed at the nodes. The sample numbers are indicated in parenthesis, and the accession numbers are placed following the names of isolates.

The results of maximum likelihood phylogenetic tree analysis conducted on *O*. *viverrini* revealed that the tree topologies of *cox1* and *nad1* nucleotide sequences mostly formed a monophyletic group, consistent with reference isolates. The phylogenetic analysis of *cox1* in *O*. *viverrini* collected from Chiang Mai (CM1–CM9) revealed that the majority of haplotypes formed monophyletic clusters and displayed a strong genetic relationship with reference isolates. Specifically, CM1, CM2, CM5, CM6, and CM9 haplotypes, obtained from Chiang Mai Province, were grouped together and shared genetic similarities with reference isolates originating from various regions, including northern Thailand (LP-1 and LP-5), northeastern Thailand (MDM-5, BR-2, MD-5, SKPK18M-3, SKNH-5, H5, HH22-1, VTNK, NP, SSK), Vietnam (BD1), and Lao PDR (JF739555). Moreover, two cat haplotypes (C4 and C24) were included in this clade, with C4 demonstrating a close genetic relationship with CM5 from Chiang Mai Province. Furthermore, CM4 and CM8 were closely related to haplotypes from northeastern Thailand (KK, NP, SSK) and Lao PDR (KM, SV-4). Additionally, CM8 obtained in this study exhibited close genetic similarity to cat haplotype C13-6, whereas haplotype CM7 was related to cat haplotypes C16 and C40-1. Moreover, haplotype CM3 displayed close genetic similarity to the reference isolate KPK18M-3 obtained from northeastern Thailand, with a bootstrap value of 77%. Cat haplotypes were clearly diverged from human haplotypes with a bootstrap value of 60%. Interestingly, haplotype SK diverged from the clade and clustered with cat haplotypes, particularly showing close genetic similarity to C51, with a bootstrap value of 77% (Fig 3A). A phylogenetic tree of *O*. *viverrini* based on *nad1* revealed that 10 haplotypes (CMNMU1–CMNMU10) were monophyletic groups with close relationships with reference isolates from Vietnam (OvPY3 and PY3) and Lao PDR (CP/14). The CMNMU6–CMNMU10 haplotypes were genetically closely related, with a bootstrap value of 82%, and CMMU4 and CMNMU5 were genetically close, with a bootstrap value of 50%, suggesting a close genetic relationship (Fig 3B).

**Fig 4:**
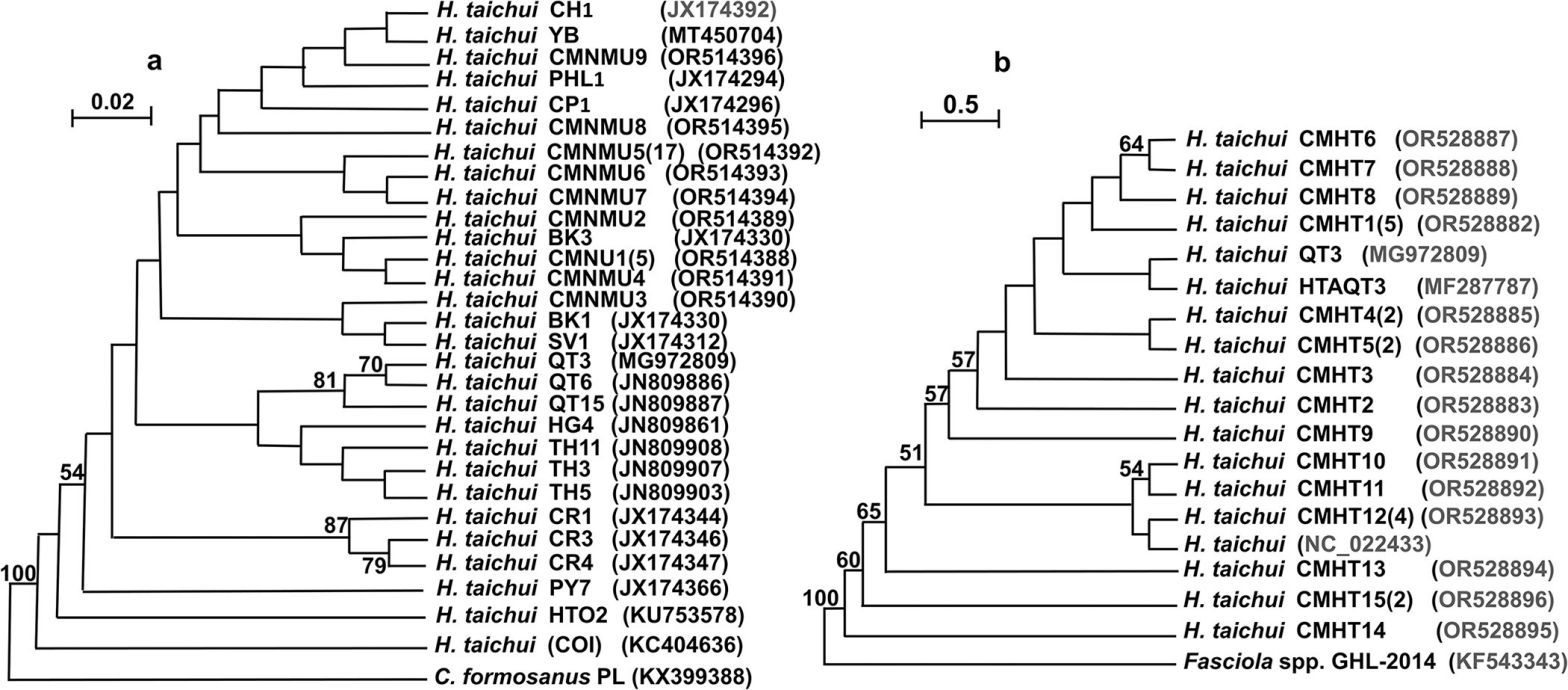
**Maximum likelihood phylogenetic trees based on *cox1* (a) and *nad1* (b) nucleotide sequences of *Haplorchis taichui*.** (a) For *cox1* tree topologies, an alignment of 375-bp nucleotide sequences with no gaps and nine haplotypes from Chiang Mai Province (CMNMU1–CMNMU9) were created. The reference of *cox1* isolates comprised isolates collected from Thailand (CR1, CR3, CR4, PY7, CH1), Lao PDR (HG4, QT3, QT6, QT15, TH3, TH5, TH11, YB, KC404636), Vietnam (BK1, BK3, SV1, CP1), and the Philippines (PHL1). (b) The phylogenetic tree of *nad1* was constructed using an alignment of 906-bp nucleotide sequences with no gaps and 15 haplotypes (CMHT1–CMHT15) collected from Chiang Mai Province. The reference isolates of *nad1* included isolates from Vietnam (HTAQT3, QT3) and Korea (NC_022433). The nodes showed 1,000 replication (bootstrap) percentages. More than 50% were displayed at the nodes. The sample numbers are indicated in parenthesis, and the accession numbers are placed following the names of isolates.

The phylogenetic tree of *H*. *taichui* using *cox1* and *nad1* nucleotide sequences from Chiang Mai Province also revealed a monophyletic group. In the phylogenetic tree of *H*. *taichui* based on *cox1*, nine haplotypes were grouped together with reference isolates from central Thailand (CH1) and Lao PDR (BK1, BK3, CP1, SV1). Moreover, haplotypes CMNU1 and CMNU4 were closely related to the reference isolate BK3, whereas haplotype CMNU1 was closely related to reference isolates BK1 and SV1 (Fig 4A). Based on *nad1* nucleotide sequences from Chiang Mai Province, the phylogenetic tree of *H*. *taichui* showed that most haplotypes were within the same taxonomic group, being monophyletic and clustering with reference isolates from Vietnam (QT3 and HTAQT3). Furthermore, haplotypes CMHT10, CMHT11, and CMHT12 were grouped together, whereas haplotypes CMHT13, CMHT14, and CMHT15 showed paraphyletic characteristics or genetic divergence from other haplotypes (Fig 4B).

### Haplotype network of *O*. *viverrini* and *H*. *taichui* based on *cox1* and *nad1* nucleotide sequences

**Fig 5:**
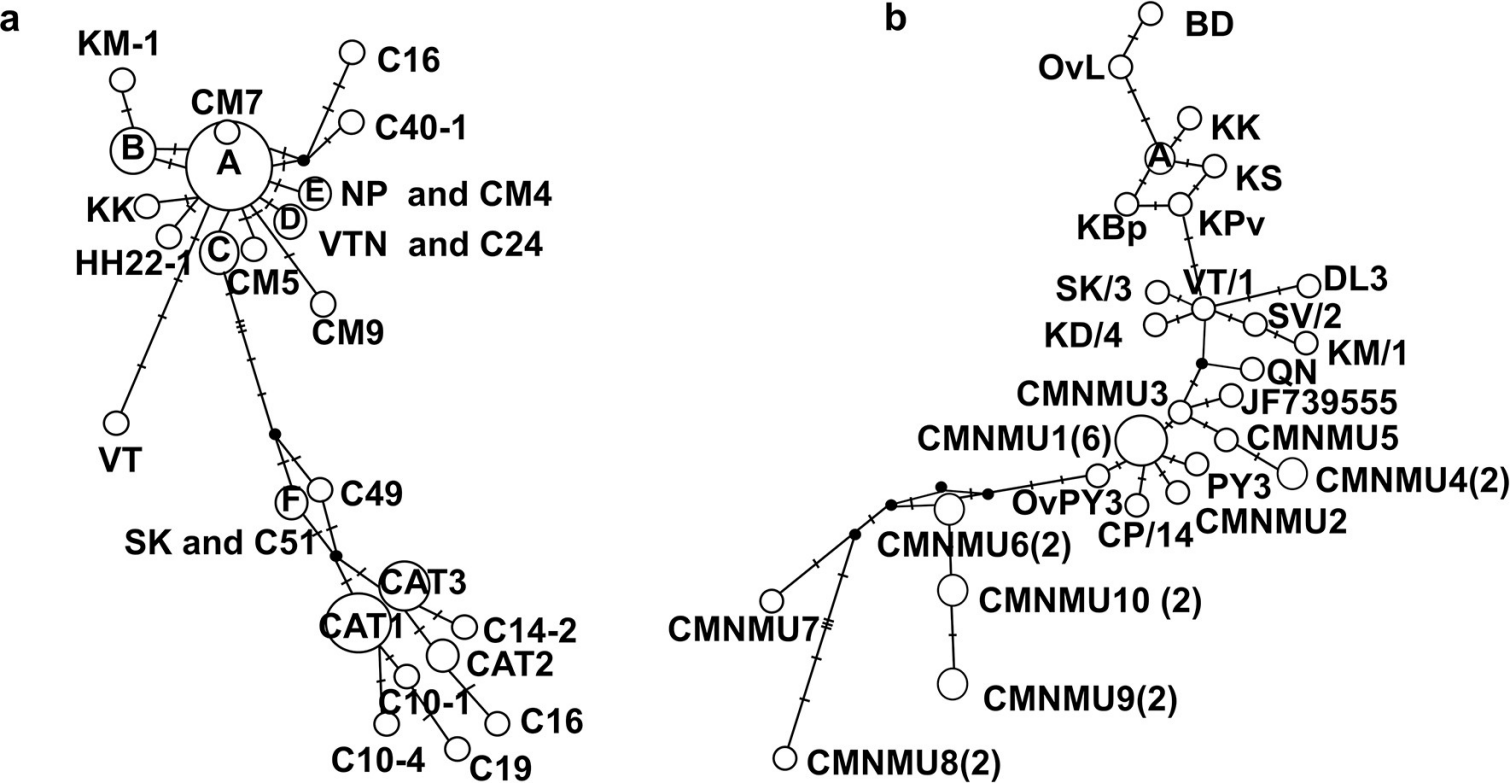
**Median-joining haplotype networks of *cox1* (a) and *nad1* (b) sequences of *Opisthorchis viverrini* retrieved from Chiang Mai Province, Thailand.** (a) The haplotype network of *cox1* was built with nine haplotypes (CM1–CM9) from Chiang Mai Province, northern Thailand and 13 reference haplotypes from Thailand (MDM-5, BR-2, MD-5, SKPK18M-3, SKNH-5, H5, HH22-1, VTNK, NP, and SSK), eastern Thailand (SK), Lao PDR (VT, KM-1, SV-4, JF739555), and Vietnam (BD1). Haplotype A comprised BD, MDM-5, BR-2, MD-5, LP-5, H5, LP-1, JF739555, SKNH-5, CM1(13), CM2, CM6(3), and C4. Haplotype B comprised SSK, SV-4, CM8(3), and C13-6. Haplotype C comprised SKPK18M-3 and CM3(2). Haplotype D comprised VTN and C24. Haplotype E comprised NP and CM4. Haplotype CAT1 comprised C3-5, C8-1, C13-2, C14-5, C21-2, C29-4, C38-2 C39-2, C40B, C43-1, C7, C25, C46, and C50. Haplotype CAT2 comprised C15B-3 and C43-2. Haplotype CAT3 comprised C14-4, C27-6, C35A-1, C38-1, C39-3, C40-3, C43-5, and C1. Other cat haplotypes included C4, C24, C13-6, C16, C40-1, C49, C10-6, C14-2, C51, C10-4, C19, and C10-1. (b) The haplotype network of *nad1* was constructed using 10 haplotypes (CMNMU1–CMNMU10) obtained from Chiang Mai Province and included 19 reference haplotypes collected from Thailand (LP-1, LP-5, KLp, KS, KBp, KPv, KK, and DQ119551), Lao PDR (VT/1, SV/2, KM/1, JF739555, CP/14), and Vietnam (BD, OvL, DL3, QN, PY3, and OvPY3) with haplotype A comprising KLp and DQ119551. The numbers of samples are placed in parenthesis.

The haplotype networks of *O*. *viverrini* based on *cox1* and *nad1* collected from Chiang Mai Province and reference isolates were constructed and showed star-like patterns, indicating a rapidly expanding population. The haplotype network of *O*. *viverrini* based on *cox1* revealed two distinct groups: human and cat haplotypes. Haplotype A emerged as the predominant and possibly ancestral haplotype. In addition, singletons (CM5, CM7, and CM9) retrieved from Chiang Mai Province exhibited genetic differences from other haplotypes (Fig 5A). Moreover, five cat haplotypes (i.e., C4, C13-6, C16, C40-1, and C24) were grouped within the cluster of human haplotypes. Haplotypes CM1, CM2, and CM6 obtained from Chiang Mai Province showed a close genetic relationship with cat haplotype C4, whereas haplotype CM8 formed a cluster with cat haplotype C13-6. Furthermore, haplotype SK was distinct from human haplotypes and categorized with cat haplotype C51. Among the cat haplotypes, haplotype CAT1 was the predominant one, comprising 14 isolates (Fig 5A).

A haplotype network of *O*. *viverrini* using *nad1* also presented population expansion. Haplotype CMNMU1 was dominant and exhibited high frequency, whereas haplotypes CMNMU6, CMNMU7, CMNMU8, CMNMU9, and CMNMU10 displayed obvious distinct genetic differences from another haplotype (Fig 5B).

**Fig 6:**
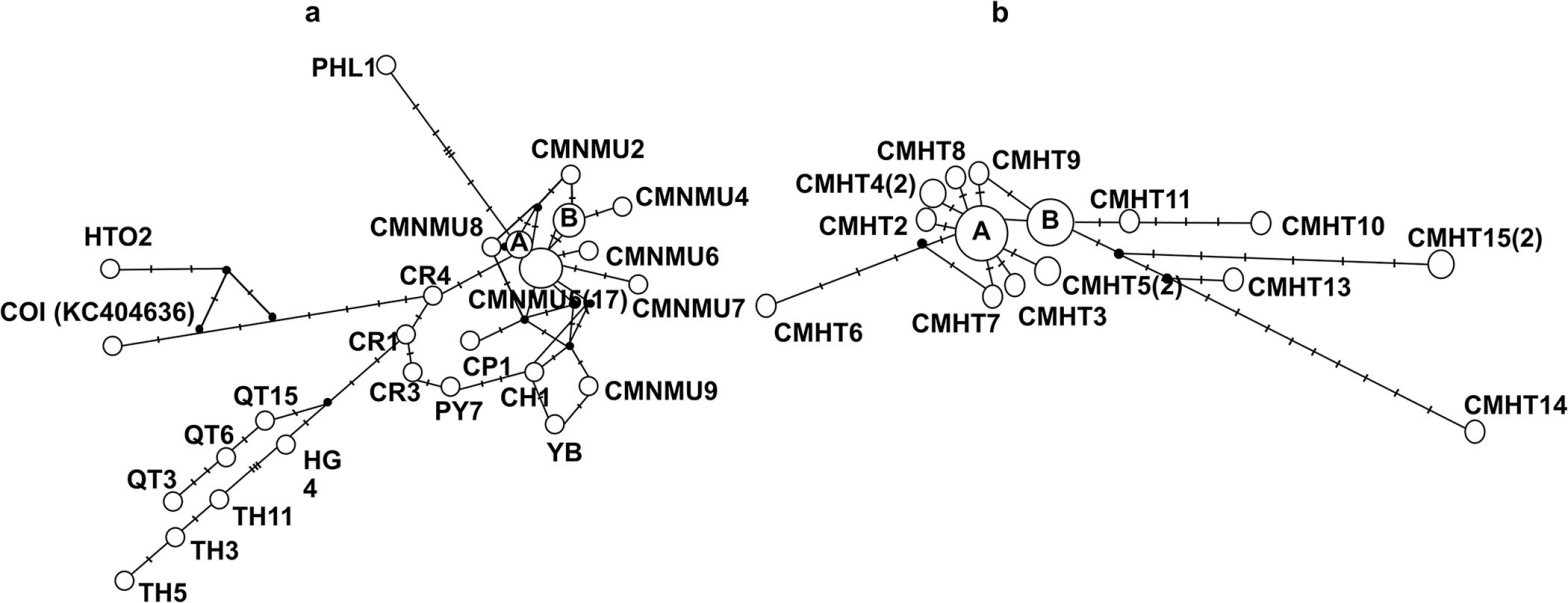
**Median-joining haplotype networks of *cox1* (a) and *nad1* (b) sequences of *Haplorchis taichui* obtained from Chiang Mai Province, Thailand.** (a) The haplotype network of *cox1* was built with nine haplotypes (CMNMU1–CMNMU9) from Chiang Mai Province, northern Thailand, and 19 reference haplotypes from Thailand (CR1, CR3, CR4, PY7, CH1), Lao PDR (HG4, QT3, QT6, QT15, TH3, TH5, TH11, YB, KC404636), Vietnam (BK1, BK3, SV1, CP1), and the Philippines (PHL1). Haplotype A comprised CMNMU3, BK1, and SV1, whereas Haplotype B comprised CMNMU1 and BK3. (b) The haplotype network of *nad1* was constructed using 15 haplotypes (CMHT1–CMHT15) obtained from Chiang Mai Province and included three reference haplotypes collected from Vietnam (HTAQT3, QT3) and Korea (NC_022433). Haplotype A comprised CMHT1, HTAQT3, and QT3, whereas Haplotype B comprised CMHT12 and NC_022433. The numbers of samples are placed in parenthesis.

The *cox1* haplotype network of *H*. *taichui* showed that haplotype CMNMU5 had a high frequency and was predominant. Haplotypes A and B and other haplotypes retrieved from Chiang Mai Province were genetically closely related. Moreover, haplotypes CMNMU2, CMNMU3, CMNMU4, CMNMU6, CMNMU7, and CMNMU8 were all singletons and demonstrated close genetic relationship to haplotypes A and B (Fig 6A). The *nad1* haplotype network of *H*. *taichui* revealed that haplotype A was dominant and possibly the ancestral haplotype. In addition, several haplotypes from Chiang Mai Province, including CMHT2, CMHT3, CMHT4, CMHT6, CMHT7, CMHT8, CMHT9, CMHT10, CMHT11, CMHT13, and CMHT14, were observed as singletons, suggesting rapid population growth. Furthermore, haplotypes CMHT11, CMHT10, CMHT15, CCMHT13, and CMHT14 showed distinct divergence patterns with increasing distance from other haplotypes, particularly CMHT14 (Fig 6B).

## Discussion

This study first investigated the genetic structure of *Opisthorchis*-like eggs in northern Thailand using mitochondrial genes, namely, *cox1* and *nad1*. The results of ITS2-PCR assay of 205 fecal samples collected from Chiang Mai Province showed the high prevalence of *H*. *taichui* (79.5% overall) and *O*. *viverrini* (31.2% overall). This finding indicates that *O*. *viverrini* plays a major role in causing CCA in northern Thailand. Because of their similar endemic areas and second intermediate host, a high prevalence of co-infections (59.0%) between *O*. *viverrini* and *H*. *taichui* was also observed, which were commonly found in northern Thailand. In addition, the findings of this study show that using a light microscope for diagnosis could lead to misdiagnosis because *O*. *viverrini* and *H*. *taichui* have similar egg morphologies.

The genetic structures of *O*. *viverrini* and *H*. *taichui* were analyzed using the *cox1* and *nad1* genes. COX1-PCR assay of *O*. *viverrini* and *H*. *taichui* showed higher sensitivity than NAD1-PCR assay. Thus, COX1-PCR assay is more suitable for studying the prevalence of *Opisthorchis*-like eggs because the sensitivity for detecting *cox1* is greater than that for *nad1*. Genetic structure analysis of *O*. *viverrini* isolated from Chiang Mai Province, northern Thailand, using mitochondrial genes demonstrated that *nad1* in *O*. *viverrini* displayed higher genetic variation than *cox1*, as supported by haplotype diversity, segregation sites, pairwise *F*_ST_ analysis, and gene flow estimation. Moreover, the genetic difference of *O*. *viverrini* using *nad1* within the intrapopulation in Chiang Mai Province was higher than that between interpopulations from different geographical endemic areas. A previous study suggested that the genetic differentiation of *nad1* in *O*. *viverrini* reduced genetic variation within interpopulations while increasing it between intrapopulation [22]. Therefore, *nad1* could be more suitable for investigating genetic variation and structure than *cox1* in *O*. *viverrini*. The genetic structure of *O*. *viverrini* in Ching Mai Province based on *cox1* revealed low genetic differentiation and high gene flow within the intrapopulation. This finding suggests that equivalent allele frequencies exist, which could prevent genetic drift despite having different geographically endemic areas. However, the genetic structures of *cox1* and *nad1* in *O*. *viverrini* were similar since genetic differentiation did not show significant differences within or between different geographical areas. The population of *O*. *viverrini* in Chiang Mai Province was expanding, and selective sweep and haplotype network patterns also showed rapid population growth. However, the results of Tajima’s *D* test were not significant, and many samples are required for further investigation. The maximum likelihood phylogenetic trees based on *cox1* and *nad1* in *O*. *viverrini* isolated from humans showed genetic monophyly and shared a genetic relationship with reference haplotypes from different geographically endemic areas. Moreover, the phylogenetic tree and haplotype network of *cox1* in *O*. *viverrini* revealed that five haplotypes (C4, C13-6, C16, C24, C40-1) isolated from cats were clustered with human haplotypes, which was similar to that in a previous study [44]. Furthermore, haplotype SK was found in the same cluster with cat haplotypes, indicating that *O*. *viverrini* infections are not strictly host-specific. Additionally, haplotypes CM1, CM2, CM6, and CM8 obtained from Chiang Mai Province could be transmitted between residents in this province and cats, which serve as reservoir hosts. Additionally, haplotypes BD, MDM-5, BR-2, MD-5, LP-5, H5, LP-1, JF739555, SKNH-5, CM1, CM2, CM6, and C4 may have carried an older and dominant allele compared with singletons. Likewise, *nad1* of *O*. *viverrini* collected from Chiang Mai Province displayed strong genetic relation to haplotypes from Vietnam (OvPY3 and PY3), implying that they might be ancient ancestral haplotypes. Recently, *O*. *viverrini* populations in Lower Mekong Basin were found to show genetic monophyly and grow without geographical barriers [22]. Gene flow plays a crucial role in the genetic structure of *O*. *viverrini* populations and is frequently influenced by the host’s movement patterns, particularly in regions with complex transmission dynamics. For example, the movement of the second intermediate host, cyprinoid fish, which typically inhabit freshwater bodies such as rivers, lakes, and ponds, is affected by factors including water currents, migration for spawning purposes, and environmental changes. Similarly, human movement, including travel and migration, can impact the spread of parasites in endemic areas, as can the movements of reservoir hosts (e.g., domestic cats and dogs), which may interact with infected cyprinoid fish or contaminated water sources. Additionally, *Bithynia* spp., the first intermediate host, usually inhabit freshwater habitats, including ponds, marshes, and slow-moving streams, and their movements are primarily confined to their aquatic environment and influenced by factors such as water flow, temperature, and habitat availability [22]. Therefore, the host’s movement, especially definitive hosts including humans and cyprinoid fish, could facilitate the spread of parasite genotypes across wide geographic areas, potentially contributing to genetic variation among *O*. *viverrini* populations.

The genetic structure of *H*. *taichui* based on *cox1* and *nad1* revealed high variation but no significant population differences. The intrapopulation differences in Chiang Mai Province are lower than the interpopulation differences in different geographically endemic areas. The genetic differences within *H*. *taichui* intrapopulation in Chiang Mai Province did not show a significant difference probably because they had similar genetic structure. Gene flow (i.e., the movement of specific alleles from geographically isolated populations) can support local adaptation. Consequently, the population of *H*. *taichui* in Chiang Mai Province, northern Thailand, showed low gene flow levels, resulting in high genetic variation within intrapopulation and low variation between interpopulations. In 2019, a study reported a significant prevalence of *Opisthorchis*-like egg infections within the hill tribe population of Chiang Mai Province, northern Thailand. The findings revealed a particularly noteworthy prevalence rate, with *H*. *taichui* and *O*. *viverrini* accounting for 75.4% and 37.2% of infections, respectively [2]. Moreover, the prevalence of *O*. *viverrini* in this province was higher than that (7.2%) reported in a previous study conducted in the lower northern region of Thailand [48]. Therefore, the genetic structure of *O*. *viverrini* in this area could be representative of *O*. *viverrini* in northern Thailand. Furthermore, the *Opisthorchis*-like eggs in this study were derived from the hill tribe population. Hence, understanding the population’s genetic structure and gene flow is important for providing epidemiological data and identifying genetic diversity and differentiation patterns across geographical areas or ecological niches. However, a limitation of this study is the use of pooled parasite eggs from infected individuals, which may lead to the presence of multiple genotypes within hosts. This contrasts with conventional population genetic studies that often utilize individual parasite stages (e.g., egg, miracidium, cercaria, metacercariae, or adult worms) for genetic analysis. Nevertheless, pooled parasite eggs from infected individuals can allow us to capture a broad parasite population in infected individuals within a geographic area or host population within the same local endemic areas. Moreover, fecal samples with pooled parasite eggs increase the likelihood of detecting rare alleles or low-frequency genetic variants within the parasite population. In this study, the number of parasite eggs recovered from each sample was not quantified, which directly impacts the amount of DNA available for genetic analysis. Insufficient DNA due to a low number of eggs can result in unsuccessful amplifications or unreliable sequencing outcomes, leading to inaccurate genetic analysis and compromising the interpretation of genetic information. Previous studies demonstrated successful PCR amplification of samples with light infections containing *Opisthorchis*-like eggs. The successful amplification can be attributed to meticulous DNA extraction protocols, optimized PCR assays, and primers with high sensitivity, which facilitate nucleotide sequencing and subsequent genetic analysis [15,49]. Additionally, samples yielding PCR-negative results may potentially limit the comprehensiveness of genetic analysis in these parasite populations. In addition, analyzing the genetic structures of parasites in fecal samples allows us to investigate intraspecific interactions and their consequences for parasite transmission and evolution in their definitive host. In this study, a subset of 30 positive samples that were randomly selected from ITS2-PCR was utilized for genetic analysis to serve as a subpopulation and represent the broader population. Subsequently, the genetic information derived from this subpopulation was analyzed to infer the genetic traits of the broader population, providing insights into their diversity, relationships, and potential for further investigation. This process involved examining patterns of genetic variation, elucidating evolutionary connections, and evaluating the effects of factors such as genetic drift and natural selection. This process involved examining genetic variation patterns, elucidating evolutionary connections, and evaluating the effects of factors such as genetic drift or natural selection. Additionally, this preliminary assessment of the genetic structure of *Opisthorchis*-like eggs in the northern region of Thailand informs decisions on whether to expand the study further or refine research methods. However, genetic analysis conducted on a subpopulation may not be entirely generalizable to the entire population. If the subpopulation does not accurately reflect the broader population, there is a risk of either overestimating or underestimating certain genetic characteristics. The genetic variation of *Opisthorchis-*like eggs in Chiang Mai Province, northern Thailand, may be influenced by several factors such as parasite lifespan, spatial distribution, random mating size, distribution of intermediate snail host population, and seasonal infective dynamics rather than geographic distance. For example, the genetic structure and expansion of *O*. *viverrini* and *H*. *taichui* in Chiang Mai Province, northern Thailand, may depend on seasonal factors. *O*. *viverrini* infections are limited to the hot–dry season, whereas *H*. *taichui* infections peak during the hot–dry season followed by a gradual decrease throughout the rainy and cool–dry season [50]. Furthermore, the lifespan of *O*. *viverrini* and *H*. *taichui* significantly influences their genetic structures as *H*. *taichui* has a shorter lifespan, which could reduce gene flow across populations. Additionally, their life cycles require intermediate hosts, leading to a population bottleneck during dry seasons. The gene flow between *O*. *viverrini* and *H*. *taichui* populations may be correlated with local adaptations and environmental changes, rather than geographic distance. However, due to the limited number of samples and localities available for investigating the genetic structure of *Opisthorchis*-like eggs in northern Thailand, further research is required. Expanding the number of localities and increasing the sample size will be crucial to accurately clarify the genetic differentiation in this region. In addition, next-generation sequencing (NGS) technologies should be utilized to conduct a more comprehensive genetic analysis and detect rare and low-frequency alleles, thereby providing a more detailed and in-depth genetic landscape of the parasite population in this endemic area. Moreover, another genetic marker, such as microsatellite loci, should be applied to reveal the population genetic structure of these parasite in this endemic area. In addition, molecular epidemiology data of intermediate hosts between seasons and reservoir hosts remain insufficient and should be examined to understand their genetic structure. The genetic structure of *Opisthorchis*-like egg parasites may also be related to clinical manifestations and pathology, particularly CCA. Thus, the information obtained in this research will contribute to control and treatment programs that may impact individuals residing in endemic areas.

In conclusion, this study is the first to examine the genetic structures of *Opisthorchis*-like eggs from hill tribe population in northern Thailand. Genetic discrimination of *Opisthorchis*-like eggs from Chaing Mai Province, northern Thailand, revealed the high prevalence of *H*. *taichui* and *O*. *viverrini* and co-infection of both parasites. This finding indicates that *O*. *viverrini* may be related to the incidence of CCA in northern Thailand. Moreover, genetic analysis of *O*. *viverrini* and *H*. *taichui* showed no significant genetic differentiation either within or between populations, suggesting that these populations are monophyletic. In addition, *H*. *taichui* and *O*. *viverrini* populations revealed population expansion and selective sweep in the study area, and both parasites displayed close genetic relationships with isolates obtained from other geographic regions, such as northern Thailand, Lao PDR, and Vietnam.

## References

[1] Radomyos B, Wongsaroj T, Wilairatana P, Radomyos P, Praevanich R, Meesomboon V, et al. Opisthorchiasis and intestinal fluke infections in northern Thailand. Southeast Asian J Trop Med Public Health. 1998;29(1):123–7. . 9740284

[2] Buathong S, Phaiphilai K, Ruang-Areerate T, Sitthichot N, Thita T, Mungthin M, et al. Genetic differentiation of *Opisthorchis*-like eggs in northern Thailand using stool specimens under national strategic plan to control liver fluke infection and cholangiocarcinoma. Am J Trop Med Hyg. 2020;103(3):1118–24. doi: 10.4269/ajtmh.20-0231 ; PubMed Central PMCID: PMC7470534. 32588808 PMC7470534

[3] Sripa B, Kaewkes S, Sithithaworn P, Mairiang E, Laha T, Smout M, et al. Liver fluke induces cholangiocarcinoma. PLoS Med. 2007;4(7):e201. doi: 10.1371/journal.pmed.0040201 ; PubMed Central PMCID: PMC1913093. 17622191 PMC1913093

[4] Kaewpitoon N, Kaewpitoon SJ, Pengsaa P, Sripa B. *Opisthorchis viverrini*: the carcinogenic human liver fluke. World J Gastroenterol. 2008;14(5):666–74. doi: 10.3748/wjg.14.666 ; PubMed Central PMCID: PMC2683991. 18205254 PMC2683991

[5] Smout MJ, Sripa B, Laha T, Mulvenna J, Gasser RB, Young ND, et al. Infection with the carcinogenic human liver fluke, *Opisthorchis viverrini*. Mol Biosyst. 2011;7(5):1367–75. Epub 20110211. doi: 10.1039/c0mb00295j ; PubMed Central PMCID: PMC3739706. 21311794 PMC3739706

[6] Sripa B, Bethony JM, Sithithaworn P, Kaewkes S, Mairiang E, Loukas A, et al. Opisthorchiasis and *Opisthorchis*-associated cholangiocarcinoma in Thailand and Laos. Acta Trop. 2011;120 Suppl 1 (Suppl 1):S158–68. Epub 20100723. doi: 10.1016/j.actatropica.2010.07.006 ; PubMed Central PMCID: PMC3010517. 20655862 PMC3010517

[7] Hughes T, O’Connor T, Techasen A, Namwat N, Loilome W, Andrews RH, et al. Opisthorchiasis and cholangiocarcinoma in Southeast Asia: an unresolved problem. Int J Gen Med. 2017;10:227–37. Epub 20170810. doi: 10.2147/IJGM.S133292 ; PubMed Central PMCID: PMC5557399. 28848361 PMC5557399

[8] Sripa B, Tangkawattana S, Brindley PJ. Update on pathogenesis of opisthorchiasis and cholangiocarcinoma. Adv Parasitol. 2018;102:97–113. Epub 20181022. doi: 10.1016/bs.apar.2018.10.001 . 30442312

[9] Vatanasapt V, Tangvoraphonkchai V, Titapant V, Pipitgool V, Viriyapap D, Sriamporn S. A high incidence of liver cancer in Khon Kaen Province, Thailand. Southeast Asian J Trop Med Public Health. 1990;21(3):489–94. . 1963706

[10] Clausen J, Murrell D. Heminth-Nematode: *Haplorchis*. In Elsevier eBooks; 2014. pp. 99–103. Available from: 10.1016/b978-0-12-378612-8.00409-1

[11] Charoensuk L, Subrungruang I, Mungthin M, Pinlaor S, Suwannahitatorn P. Comparison of stool examination techniques to detect *Opisthorchis viverrini* in low intensity infection. Acta Trop. 2019;191:13–6. Epub 20181211. doi: 10.1016/j.actatropica.2018.12.018 . 30550733

[12] Kopolrat KY, Singthong S, Khuntikeo N, Loilome W, Worasith C, Homwong C, et al. Performance of Mini Parasep((R)) SF stool concentrator kit, Kato-Katz, and formalin-ethyl acetate concentration methods for diagnosis of opisthorchiasis in Northeast Thailand. Parasit Vectors. 2022;15(1):234. Epub 20220627. doi: 10.1186/s13071-022-05338-z ; PubMed Central PMCID: PMC9235228. 35761311 PMC9235228

[13] Tesana S, Srisawangwonk T, Kaewkes S, Sithithaworn P, Kanla P, Arunyanart C. Eggshell morphology of the small eggs of human trematodes in Thailand. Southeast Asian J Trop Med Public Health. 1991;22(4):631–6. . 1820654

[14] Lovis L, Mak TK, Phongluxa K, Soukhathammavong P, Sayasone S, Akkhavong K, et al. PCR Diagnosis of *Opisthorchis viverrini* and *Haplorchis taichui* Infections in a Lao Community in an area of endemicity and comparison of diagnostic methods for parasitological field surveys. J Clin Microbiol. 2009;47(5):1517–23. Epub 20090311. doi: 10.1128/JCM.02011-08 ; PubMed Central PMCID: PMC2681877. 19279176 PMC2681877

[15] Buathong S, Leelayoova S, Mungthin M, Ruang-Areerate T, Naaglor T, Suwannahitatorn P, et al. Molecular discrimination of *Opisthorchis*-like eggs from residents in a rural community of central Thailand. PLoS Negl Trop Dis. 2017;11(11):e0006030. Epub 20171102. doi: 10.1371/journal.pntd.0006030 ; PubMed Central PMCID: PMC5685638. 29095828 PMC5685638

[16] Traub RJ, Macaranas J, Mungthin M, Leelayoova S, Cribb T, Murrell KD, et al. A new PCR-based approach indicates the range of *Clonorchis sinensis* now extends to Central Thailand. PLoS Negl Trop Dis. 2009;3(1):e367. Epub 20090120. doi: 10.1371/journal.pntd.0000367 ; PubMed Central PMCID: PMC2614470. 19156191 PMC2614470

[17] Pusadee Sri-aroon PB, Jareemate Limsoomboon, Manus Kaewpoolsri, Yupa Chusongsang, Prasasana Charoenjai, Phiraphol Chusongsang, Suthep Numnuan, Songtham Kiatsiri. Freshwater mollusks at designated areas in eleven provinces of Thailand according to the water resource development projects. Southeast Asian J Trop Med Public Health. 2007;38(2):294–301. 17539279

[18] Sato M, Thaenkham U, Dekumyoy P, Waikagul J. Discrimination of *O*. *viverrini*, *C*. *sinensis*, *H*. *pumilio* and *H*. *taichui* using nuclear DNA-based PCR targeting ribosomal DNA ITS regions. Acta Trop. 2009;109(1):81–3. Epub 20081004. doi: 10.1016/j.actatropica.2008.09.015 . 18952037

[19] Pumpa S, Phadungsil W, Grams R, Martviset P, Ruang-Areerate T, Mungthin M, et al. Improvement of a PCR-based method for the detection of *Opisthorchis viverrini* eggs in human fecal samples by targeting internal transcribed spacer-2 (ITS-2), cytochrome oxidase subunit 1 (*cox1*), and cytochrome b (*cyb*). J Parasit Dis. 2021;45(2):474–8. Epub 20210102. doi: 10.1007/s12639-020-01329-y ; PubMed Central PMCID: PMC8254682. 34295047 PMC8254682

[20] Thaenkham U, Visetsuk K, Dung do T, Waikagul J. Discrimination of *Opisthorchis viverrin*i from *Haplorchis taichui* using COI sequence marker. Acta Trop. 2007;103(1):26–32. Epub 20070518. doi: 10.1016/j.actatropica.2007.05.006 . 17574199

[21] Buathong S, Leelayoova S, Mungthin M, Naaglor T, Taamasri P, Suwannahitatorn P, et al. Development and evaluation of PCR methods based on cytochrome c oxidase subunit one (*cox1*) and NADH dehydrogenase subunit one gene (*nad1*) to detect *Opisthorchis viverrini* in human fecal samples. Parasitol Res. 2015;114(9):3547–9. Epub 20150805. doi: 10.1007/s00436-015-4640-7 . 26239798

[22] Thaenkham U, Nuamtanong S, Sa-nguankiat S, Yoonuan T, Touch S, Manivong K, et al. Monophyly of *Opisthorchis viverrini* populations in the lower Mekong Basin, using mitochondrial DNA *nad1* gene as the marker. Parasitol Int. 2010;59(2):242–7. Epub 20100301. doi: 10.1016/j.parint.2010.02.009 . 20197110

[23] Sanpool O, Intapan PM, Thanchomnang T, Janwan P, Lulitanond V, Doanh PN, et al. Rapid detection and differentiation of *Clonorchis sinensis* and *Opisthorchis viverrini* eggs in human fecal samples using a duplex real-time fluorescence resonance energy transfer PCR and melting curve analysis. Parasitol Res. 2012;111(1):89–96. Epub 20120113. doi: 10.1007/s00436-011-2804-7 . 22246366

[24] Kaewkong W, Intapan PM, Sanpool O, Janwan P, Thanchomnang T, Laummaunwai P, et al. Molecular differentiation of *Opisthorchis viverrini* and *Clonorchis sinensis* eggs by multiplex real-time PCR with high resolution melting analysis. Korean J Parasitol. 2013;51(6):689–94. Epub 20131231. doi: 10.3347/kjp.2013.51.6.689 ; PubMed Central PMCID: PMC3916459. 24516275 PMC3916459

[25] Saijuntha W, Sithithaworn P, Wongkham S, Laha T, Pipitgool V, Petney TN, et al. Genetic markers for the identification and characterization of *Opisthorchis viverrini*, a medically important food borne trematode in Southeast Asia. Acta Trop. 2006;100(3):246–51. Epub 20061212. doi: 10.1016/j.actatropica.2006.11.001 ; PubMed Central PMCID: PMC2396402. 17166476 PMC2396402

[26] Saijuntha W, Sithithaworn P, Wongkham S, Laha T, Pipitgool V, Tesana S, et al. Evidence of a species complex within the food-borne trematode *Opisthorchis viverrini* and possible co-evolution with their first intermediate hosts. Int J Parasitol. 2007;37(6):695–703. Epub 20061229. doi: 10.1016/j.ijpara.2006.12.008 ; PubMed Central PMCID: PMC2150547. 17275001 PMC2150547

[27] Sithithaworn P, Nuchjungreed C, Srisawangwong T, Ando K, Petney TN, Chilton NB, et al. Genetic variation in *Opisthorchis viverrini* (Trematoda: Opisthorchiidae) from northeast Thailand and Laos PDR based on random amplified polymorphic DNA analyses. Parasitol Res. 2007;100(3):613–7. Epub 20061003. doi: 10.1007/s00436-006-0304-y ; PubMed Central PMCID: PMC2150546. 17016722 PMC2150546

[28] Wongsawad C, Wongsawad P. *Opisthorchis viverrini* and *Haplorchis taichui*: development of a multiplex PCR assay for their detection and differentiation using specific primers derived from HAT-RAPD. Exp Parasitol. 2012;132(2):237–42. Epub 20120801. doi: 10.1016/j.exppara.2012.07.007 . 22874527

[29] Laoprom N, Sithithaworn P, Andrews RH, Ando K, Laha T, Klinbunga S, et al. Population genetic structuring in *Opisthorchis viverrini* over various spatial scales in Thailand and Lao PDR. PLoS Negl Trop Dis. 2012;6(11):e1906. Epub 20121115. doi: 10.1371/journal.pntd.0001906 ; PubMed Central PMCID: PMC3499411. 23166853 PMC3499411

[30] Namsanor J, Pitaksakulrat O, Kopolrat K, Kiatsopit N, Webster BL, Gower CM, et al. Impact of geography and time on genetic clusters of *Opisthorchis viverrini* identified by microsatellite and mitochondrial DNA analysis. Int J Parasitol. 2020;50(14):1133–44. Epub 20200829. doi: 10.1016/j.ijpara.2020.06.011 . 32866491

[31] Saijuntha W, Sithithaworn P, Wongkham S, Laha T, Chilton NB, Petney TN, et al. Mitochondrial DNA sequence variation among geographical isolates of *Opisthorchis viverrini* in Thailand and Lao PDR, and phylogenetic relationships with other trematodes. Parasitology. 2008;135(12):1479–86. doi: 10.1017/S0031182008005015 ; PubMed Central PMCID: PMC2582335. 18937886 PMC2582335

[32] Alvi MA, Khalid A, Ali RMA, Saqib M, Qamar W, Li L, et al. Genetic variation and population structure of *Fasciola hepatica*: an in silico analysis. Parasitol Res. 2023;122(9):2155–73. Epub 20230717. doi: 10.1007/s00436-023-07917-0 . 37458821

[33] Elliott T, Muller A, Brockwell Y, Murphy N, Grillo V, Toet HM, et al. Evidence for high genetic diversity of NAD1 and COX1 mitochondrial haplotypes among triclabendazole resistant and susceptible populations and field isolates of *Fasciola hepatica* (liver fluke) in Australia. Vet Parasitol. 2014;200(1–2):90–6. Epub 20131201. doi: 10.1016/j.vetpar.2013.11.019 . 24360656

[34] Blair D, Agatsuma T, Watanobe T, Okamoto M, Ito A. Geographical genetic structure within the human lung fluke, *Paragonimus westermani*, detected from DNA sequences. Parasitology. 1997;115 (Pt 4):411–7. doi: 10.1017/s0031182097001534 . 9364568

[35] Agatsuma T, Iwagami M, Sato Y, Iwashita J, Hong SJ, Kang SY, et al. The origin of the triploid in *Paragonimus westermani* on the basis of variable regions in the mitochondrial DNA. J Helminthol. 2003;77(4):279–85. doi: 10.1079/joh2003185 . 14627442

[36] Zhao GH, Mo XH, Zou FC, Li J, Weng YB, Lin RQ, et al. Genetic variability among *Schistosoma japonicum* isolates from different endemic regions in China revealed by sequences of three mitochondrial DNA genes. Vet Parasitol. 2009;162(1–2):67–74. Epub 20090226. doi: 10.1016/j.vetpar.2009.02.022 . 19303214

[37] Yin M, Li H, McManus DP, Blair D, Su J, Yang Z, et al. Geographical genetic structure of *Schistosoma japonicum* revealed by analysis of mitochondrial DNA and microsatellite markers. Parasit Vectors. 2015;8:150. Epub 20150308. doi: 10.1186/s13071-015-0757-x ; PubMed Central PMCID: PMC4372230. 25881113 PMC4372230

[38] Webster BL, Emery AM, Webster JP, Gouvras A, Garba A, Diaw O, et al. Genetic diversity within S*chistosoma haematobium*: DNA barcoding reveals two distinct groups. PLoS Negl Trop Dis. 2012;6(10):e1882. Epub 20121025. doi: 10.1371/journal.pntd.0001882 ; PubMed Central PMCID: PMC3493392. 23145200 PMC3493392

[39] Wijit A, Morakote N, Klinchid J. High prevalence of haplorchiasis in Nan and Lampang provinces, Thailand, proven by adult worm recovery from suspected opisthorchiasis cases. Korean J Parasitol. 2013;51(6):767–9. Epub 20131231. doi: 10.3347/kjp.2013.51.6.767 ; PubMed Central PMCID: PMC3916473. 24516289 PMC3916473

[40] Aguettant JL. Impact of population registration on hilltribe development in Thailand. Asia Pac Popul J. 1996;11(4):47–72. . 12347778

[41] Kumar S, Stecher G, Li M, Knyaz C, Tamura K. MEGA X: Molecular Evolutionary Genetics Analysis across Computing Platforms. Mol Biol Evol. 2018;35(6):1547–9. doi: 10.1093/molbev/msy096 ; PubMed Central PMCID: PMC5967553. 29722887 PMC5967553

[42] Librado P, Rozas J. DnaSP v5: a software for comprehensive analysis of DNA polymorphism data. Bioinformatics. 2009;25(11):1451–2. Epub 20090403. doi: 10.1093/bioinformatics/btp187 . 19346325

[43] Slatkin M. Gene flow and the geographic structure of natural populations. Science. 1987;236(4803):787–92. doi: 10.1126/science.3576198 . 3576198

[44] Sota P, Suttiprapa S, Tangkawattana S, Sripa M, Blair D, Sripa B. Does *Opisthorchis viverrini* circulate between humans and domestic cats in an endemic area in Thailand? Parasitology. 2022;149(10):1334–8. Epub 20220510. doi: 10.1017/S0031182022000646 . 35535483 PMC11010474

[45] Excoffier L, Laval G, Schneider S. Arlequin (version 3.0): an integrated software package for population genetics data analysis. Evol Bioinform Online. 2007;1:47–50. Epub 20070223. ; PubMed Central PMCID: PMC2658868. 19325852 PMC2658868

[46] Tamura K. Estimation of the number of nucleotide substitutions when there are strong transition-transversion and G+C-content biases. Mol Biol Evol. 1992;9 (4):678–87. doi: 10.1093/oxfordjournals.molbev.a040752 . 1630306

[47] Fu YX. Statistical tests of neutrality of mutations against population growth, hitchhiking and background selection. Genetics. 1997;147(2):915–25. doi: 10.1093/genetics/147.2.915 ; PubMed Central PMCID: PMC1208208. 9335623 PMC1208208

[48] Pumidonming W, Katahira H, Igarashi M, Salman D, Abdelbaset AE, Sangkaeo K. Potential risk of a liver fluke *Opisthorchis viverrini* infection brought by immigrants from prevalent areas: A case study in the lower Northern Thailand. Acta Trop. 2018;178:213–8. Epub 20171206. doi: 10.1016/j.actatropica.2017.11.023 . 29191517

[49] Boondit J, Suwannahitatorn P, Siripattanapipong S, Leelayoova S, Mungthin M, Tan-Ariya P, et al. An epidemiological survey of *Opisthorchis viverrini* infection in a lightly infected community, eastern Thailand. Am J Trop Med Hyg. 2020;102(4):838–43. doi: 10.4269/ajtmh.19-0864 ; PubMed Central PMCID: PMC7124929. 32043456 PMC7124929

[50] Wongsawad C, Phalee A, Noikong W, Chuboon S, Nithikathkul C. Co-infection with *Opisthorchis viverrini* and *Haplorchis taichui* detected by human fecal examination in Chomtong district, Chiang Mai Province, Thailand. Parasitol Int. 2012;61(1):56–9. Epub 20111025. doi: 10.1016/j.parint.2011.10.003 . 22047704

