# Correction: Study of the population genetic structure of *Opisthorchis*-like eggs in northern Thailand using mitochondrial genes

**DOI:** 10.1371/journal.pntd.0013173

**Published:** 2025-06-04

**Authors:** Picha Suwannahitatorn, Mathirut Mungthin, Ittisak Subrungruang, Lakhanawan Charoensuk, Nithikoon Aksorn, Saiwasan Buathong

There are a number of errors in the caption for Fig 4 “Maximum likelihood phylogenetic trees based on cox1 (a) and nad1 (b) nucleotide sequences of Haplorchis taichui” and Fig 6 “Median-joining haplotype networks of cox1 (a) and nad1 (b) sequences of Haplorchis taichui obtained from Chiang Mai Province, Thailand”. Please see the complete, correct Fig 4 and Fig 6 captions here.

**Fig 4:**
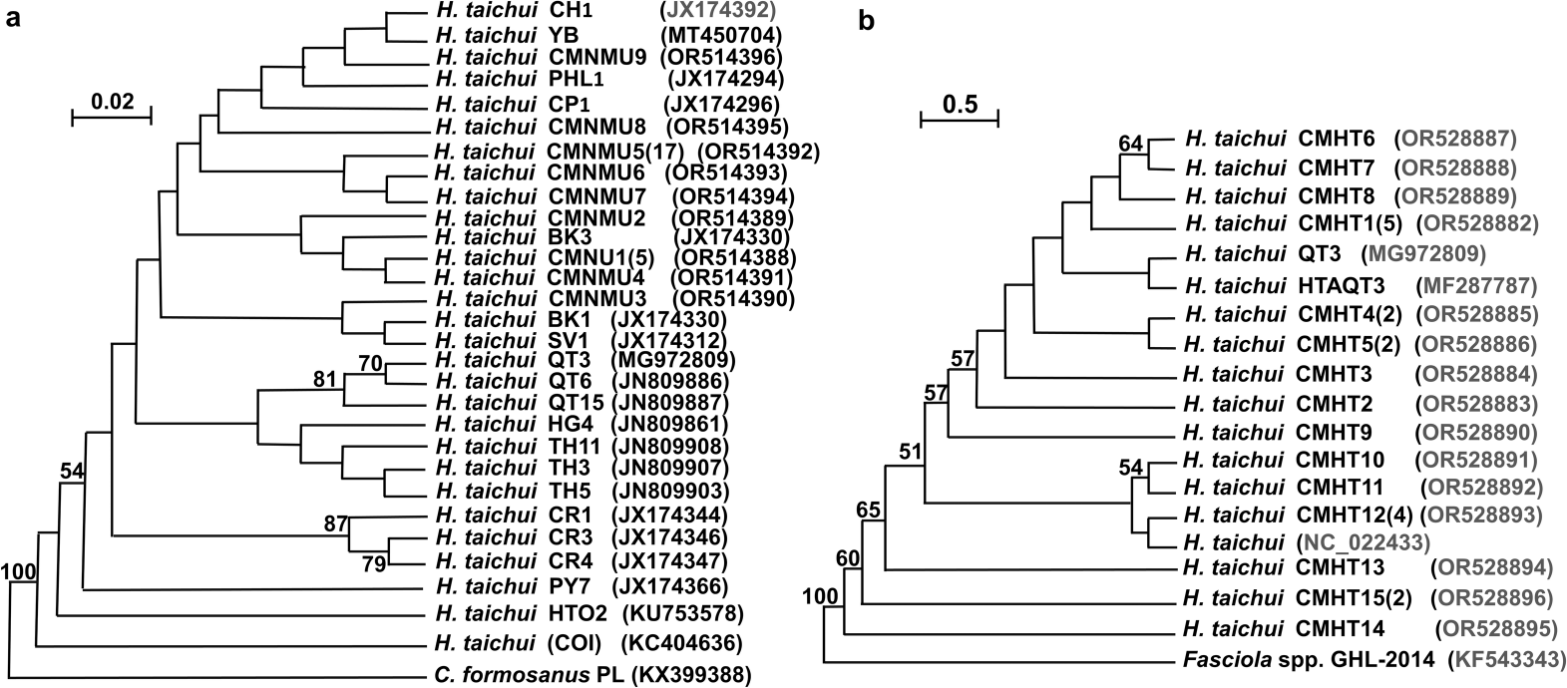
Maximum likelihood phylogenetic trees based on *cox1* (a) and *nad1* (b) nucleotide sequences of *Haplorchis taichui.* **(a)** For *cox1* tree topologies, an alignment of 375-bp nucleotide sequences with no gaps and nine haplotypes from Chiang Mai Province (CMNMU1–CMNMU9) were created. The reference of *cox1* isolates comprised isolates collected from Thailand (CR1, CR3, CR4, PY7, CH1), Vietnam (HG4, QT3, QT6, QT15, TH3, TH5, TH11, YB, KC404636), Lao PDR (BK1, BK3, SV1, CP1), and the Philippines (PHL1). **(b)** The phylogenetic tree of *nad1* was constructed using an alignment of 906-bp nucleotide sequences with no gaps and 15 haplotypes (CMHT1–CMHT15) collected from Chiang Mai Province. The reference isolates of *nad1* included isolates from Vietnam (HTAQT3, QT3) and Korea (NC_022433). The nodes showed 1,000 replication (bootstrap) percentages. More than 50% were displayed at the nodes. The sample numbers are indicated in parenthesis, and the accession numbers are placed following the names of isolates.

**Fig 6:**
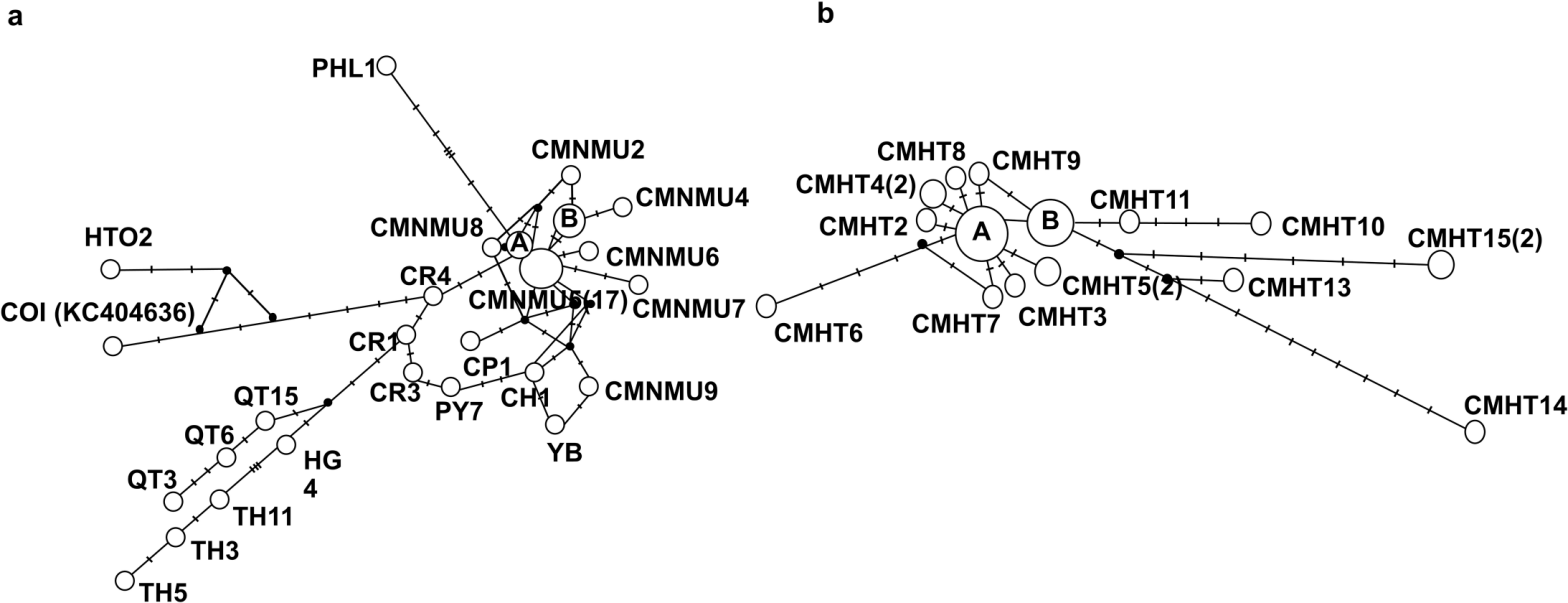
Median-joining haplotype networks of *cox1* (a) and *nad1* (b) sequences of *Haplorchis taichui* obtained from Chiang Mai Province, Thailand. **(a)** The haplotype network of *cox1* was built with nine haplotypes (CMNMU1–CMNMU9) from Chiang Mai Province, northern Thailand, and 19 reference haplotypes from Thailand (CR1, CR3, CR4, PY7, CH1), Vietnam (HG4, QT3, QT6, QT15, TH3, TH5, TH11, YB, KC404636), Lao PDR (BK1, BK3, SV1, CP1), and the Philippines (PHL1). Haplotype A comprised CMNMU3, BK1, and SV1, whereas Haplotype B comprised CMNMU1 and BK3. **(b)** The haplotype network of *nad1* was constructed using 15 haplotypes (CMHT1–CMHT15) obtained from Chiang Mai Province and included three reference haplotypes collected from Vietnam (HTAQT3, QT3) and Korea (NC_022433). Haplotype A comprised CMHT1, HTAQT3, and QT3, whereas Haplotype B comprised CMHT12 and NC_022433. The numbers of samples are placed in parenthesis.
